# Effects of Grapevine Leafroll-Associated Virus 3 on the Chemical and Sensory Properties of Cabernet Sauvignon Grape and Wine

**DOI:** 10.3390/foods15040624

**Published:** 2026-02-09

**Authors:** Na Liu, Wenguang Jiang, Huixuan Zhou, Xinyi Hao, Guotian Liu, Wenwu Bao, Xinming Zhou, Tengfei Xu, Yan Xu

**Affiliations:** 1 College of Horticulture, Northwest A&F University, Yangling, Xianyang 712100, China; liuna0403@nwafu.edu.cn (N.L.); wenwubao@nwafu.edu.cn (W.B.); 2 State Key Laboratory of Crop Stress Resistance and High-Efficiency Production, Northwest A&F University, Yangling, Xianyang 712100, China; 3 Key Laboratory of Horticultural Plant Biology and Germplasm Innovation in Northwest China, Ministry of Agriculture, Northwest A&F University, Yangling, Xianyang 712100, China; 4 Ningxia Changyu Longyu Estate Co., Ltd., Yinchuan 750000, China; zyjiangwenguang@163.com (W.J.); 13589764546@163.com (X.Z.); 5 College of Enology, Northwest A&F University, Yangling, Xianyang 712100, China; 6 School of Enology & Horticulture, Ningxia University, Yinchuan 750021, China; xinyihao@nxu.edu.cn

**Keywords:** grapevine leaf roll-associated virus, cabernet sauvignon, volatile compound, phenolic compound

## Abstract

Grapevine leafroll-associated virus (GLRaV) is a globally widespread disease that causes substantial economic losses in the wine industry. In severely affected vineyards, GLRaV can reduce grape yield by 20–40%. This study aimed to evaluate the effects of GLRaV infection on polyphenolic and volatile organic compound contents, as well as on the sensory profiles of the resulting wines. A comparative analysis was conducted between GLRaV-3-infected and healthy Cabernet Sauvignon grapes and their corresponding wines. Results show GLRaV-3 significantly alters grape and wine quality. In infected grapes, sugar content decreased while titratable acidity increased. Polyphenol composition was notably altered: phenolic acids and flavonols increased by 22.46% and 15.27%, respectively, whereas flavanols decreased by 17.86%. The levels of aldehydes and C_6_ compounds also rose significantly in the berries. Wines produced from infected grapes showed lower alcohol content and reduced dry extract. Phenolic acids and flavanols were decreased, but total flavonols increased by 12.54%. Among volatile compounds, alcohols, phenols, and fatty acids were elevated, while esters declined by 13.36%. These chemical changes directly influenced sensory attributes. Compared with wines from healthy grapes, those from infected grapes exhibited improved tannin texture and longer aftertaste. However, they were inferior in color intensity, aroma intensity, body fullness, and varietal typicity.

## 1. Introduction

Grapevine leaf roll-associated virus (GLRaV) significantly affects wine grape and wine quality. Once infected, a grapevine carries the virus for life, causing persistent harm. Moreover, chemical pesticides cannot effectively control it. GLRaVs are widely distributed in China, with more severe incidence reported in Beijing, Hebei, Tianjin, Shandong, and Ningxia [1,2,3]. Six types of GLRaVs have been detected worldwide, including GLRaV-1, GLRaV-2, GLRaV-3, and GLRaV-4 (which has variants such as GLRaV-5, -6, -9, -Pr, -De, and -Car), GLRaV-7, and GLRaV-13; these viruses belong to the family Closterovoridae [4,5]. Among these strains, GLRaV-3 has the highest prevalence worldwide. It is a positive single-stranded RNA virus and a member of the genus *Ampelovirus*. It is graft-transmissible and is spread by infected planting material and vector transmission by mealybugs (Homoptera: Pseudococcidae) and/or soft-scale insects (Homoptera: Coccidae) [6,7,8]. The key to preventing this virus lies in selecting tested and healthy seedlings, strictly controlling pest populations, and enhancing orchard sanitation and monitoring [3,8].

*Vitis vinifera* L. is an important wine grape species found globally. It is highly susceptible to GLRaV-3, such as the varieties of Cabernet Gernischt and Cabernet Sauvignon [3,7]. Some studies on GLRaV-3 have revealed its multifaceted effects on grape yield, fruit quality, wine chemical composition, and sensory characteristics [2,9,10,11,12,13]. GLRaV-3 can significantly reduce grape yield. Infected grapevines have lower shoot numbers and overall weight compared to healthy, symptom-free vines, resulting in fewer fruits [9,10]. Moreover, the effects on the quality of grapes are mainly reflected in the following aspects: (1) The sugar content, especially the contents of sucrose and starch, in grapes infected with the virus decreases significantly. GLRaV-3 reduces the transportation and accumulation of sugars in leaves by damaging the phloem structure of the leaves [2,7,11,12]. It also decreases the chlorophyll content and photosynthetic rate of grape leaves [1,14]. (2) The acidity of the infected fruits increases, especially titratable acidity (TA). These changes may occur because of the interference of the virus with the sugar metabolism pathway [9]. (3) The content of anthocyanins in the fruit skin decreases significantly, affecting the color and appearance of the fruits [11].

GLRaV-3 not only affects the quality of the fruit but also indirectly affects the quality of wine by altering the chemical composition of grapes. Wine made from virus-infected grapes exhibits reduced alcohol content, lighter color, diminished fruity aromas, and increased herbal and medicinal notes. It also has a lighter body, elevated acidity, and enhanced tannin perception [9,12]. For example, the color intensity and astringency of wine made from Dolcetto grapes infected with GLRaV-3 are significantly affected [8]. The Cabernet Gernischt wine made from infected grapes has a distinct aroma found in traditional Chinese medicine. In contrast, the wine made from uninfected grapes has a distinct green smell, which is characteristic of wines of this variety [12].This leads to muted wine flavor and a tendency toward “sensory quality decline”, thereby undermining the product’s commercial value. Nevertheless, the physicochemical parameters remain within the scope permitted by relevant regulations, with no occurrences of non-compliance or exceedance of specified limits.

Most studies have focused on GLRaVs, including the genetic diversity and phylogenetic relationships of GLRaV-3, improvements in detection methods, and effects on the quality of grapes [2,3,15]. Few studies have simultaneously reported the effects of GLRaV-3 on the aroma and phenolic compounds of grapes and wines. This study provides a comprehensive and simultaneous analysis of GLRaV-3-induced alterations in phenolic and volatile compound profiles. The analysis covers the entire grape-to-wine continuum of Cabernet Sauvignon, a globally significant cultivar, within a major Chinese wine-producing region. By elucidating these virus-related metabolic changes, the research provides a scientific foundation for refining vineyard management and adapting winemaking practices to mitigate quality variations. Ultimately, this work supports viticulturists, enologists, and vineyard managers in addressing the impacts of leafroll disease, promoting sustainable viticulture and enhancing economic resilience in affected vineyards.

## 2. Materials and Methods

### 2.1. Grape Samples and Virus Detection

#### 2.1.1. Grapevine Sample Preparation

The tested grapes included two types of tissue-cultured seedlings of Cabernet Sauvignon, specifically clone 337 (ENTAV-INRA 337), a Eurasian wine grape cultivar: virus-free seedlings and seedlings harboring only Grapevine leafroll-associated virus 3 (GLRaV-3). Their mother plants originated from the eastern foot of Helan Mountain, Ningxia (38°14′ N, 106°00′ E). In spring 2021, the two groups of tissue-cultured seedlings (after one-year in vitro culture) were separately transplanted to a controlled greenhouse for acclimatization and cultivation. All growth conditions (e.g., temperature, light, humidity, and nutrient supply) were kept consistent throughout the study. The following measures were adopted for both groups of plants. A uniform substrate formula (peat: vermiculite: perlite = 3:1:1) was used. An intelligent drip irrigation system (irrigation amount: 25 ± 2 L/plant/week) was implemented. Light and temperature were regulated (day/night temperature: 25 ± 1 °C/18 ± 1 °C, photoperiod: 14 h of light and 10 h of darkness). The standardized management of fertilizer and water involved the application of a 15-10-20 N-P-K fertilizer. The EC value (Electrical Conductivity) of 1.8 ± 0.2 mS/cm reflects the content of soluble salts in the soil. No chemical pesticides were used. To ensure optimal light distribution and facilitate canopy management, all grapevines were maintained under a Vertical Shoot Positioning (VSP) system within the greenhouse. Vine balance was regulated through winter pruning, with approximately 36–48 buds retained per plant. During the spring of the 2024 growing season, shoot thinning was carried out to achieve a target density of 15–20 fruiting shoots per linear meter of trellis. These viticultural operations were consistently applied to both the disease (DG) and control (CK) groups. The plants were 4 years old at the time of harvest. Grapes were harvested on a single, commercially determined date (29 September 2024), when key winemaking indices (e.g., sugar, tannin) were stable and deemed suitable for winemaking. This common harvest date enabled a direct comparison of grape and wine quality from infected and healthy vines under standardized practical conditions. Following harvest, 1 kg of grapes was immediately snap-frozen in liquid nitrogen and stored at −40 °C for the analysis of polyphenols and volatile compounds, while 20 kg of grapes was directly used for winemaking and the determination of physicochemical parameters.

#### 2.1.2. GLRaV-3 Detection

A total of 16 plants infected with GLRaV-3 were selected as the disease group (DG), and 18 healthy plants of this batch formed the control group (CK). The screening criteria for diseased plants were as follows: leaves showing downward curling, mesophyll tissue presenting red symptoms, and leaf veins maintaining a normal green appearance (Figure S1a). The detection of GLRaV-3 was performed by conventional RT-PCR with specific primers designed from the conserved region of the GLRaV-3 HPS70 gene (Primer sequence: F: 5′-CGCTAGGGCTGTGGAAGTATT-3′ R: 5′-GTTGTCCCGGGTACCAGATAT-3′). A modified cetyltrimethylammonium bromide (CTAB) protocol was employed to isolate total RNA from 0.1 g of leaf tissue [16]. The extracted total RNA was dissolved in 30 µL of RNase-free water (Thermo Fisher Scientific, Waltham, MA, USA) and stored at −80 °C for subsequent use. First-strand cDNA was synthesized from 1 µg of total RNA in a 20 µL reaction volume using the Innova UScript II First-Strand cDNA Synthesis SuperMix (including gDNA removal reagent; Innovagene Biotech, Nanjing, China). PCR amplification was performed in a 50 µL reaction mixture containing 25 µL of MonAmp 2× Taq Mix (with dye; Monad Biotech, Wuhan, China), 2 µL of cDNA template, 3 µL of a primer mix (10 µM each of forward and reverse primers), and 20 µL of double-distilled water (Millipore, Sigma, Burlington, MA, USA). Amplification was performed in a T100 Thermal Cycler (Bio-Rad, Hercules, CA, USA) under the following conditions: at 94 °C for 5 min, followed by 35 cycles of denaturation at 94 °C for 30 s, annealing at 54 °C for 30 s, and extension at 72 °C for 15 s, with a final extension at 72 °C for 5 min. PCR products were analyzed by agarose gel electrophoresis (1.5% agarose in 1× TAE buffer) using a GelDoc XR+ Imaging System (Bio-Rad, Hercules, CA, USA). Samples displaying a band at the expected position were confirmed as positive for GLRaV-3 (Figure S1b). Prior to analysis, all grape samples were visually inspected and showed no signs of infection by Botrytis cinerea.

### 2.2. Chemicals and Standards

Chromatography-grade anhydrous ethanol was purchased from Aladdin Reagent Co., Ltd. (Shanghai, China). Hydrochloric acid was purchased from Miou Reagent Co., Ltd. (Tianjin, China), and the monomeric phenolic compound standard (chromatographic purity) was obtained from the National Institutes of Food and Drug Control (Beijing, China). Formic acid, methanol, acetonitrile (chromatographic purity), and ethanol (chromatographic grade) were purchased from Fisher Corporation, Pittsburgh, PA, USA. Na_2_HPO_4_, NaCl, and citric acid were obtained from Sinopharm Chemical Reagent Co., Ltd. (Shanghai, China).

All volatile compound standards, the internal standard of 4-methyl-1-pentanol and C_6_–C_20_ normal alkanes, were purchased from Sigma-Aldrich, St. Louis, MO, USA (purity, >90%). Monomeric phenol standards, including 12 phenolic acids, five flavanols, and seven flavonols, were purchased from the National Institutes for Food and Drug Control, Beijing, China (purity: >95%).

Pectinase, active dry wine yeast, potassium metabisulfite, and lactic acid bacteria were purchased from Lallemand Company, Toulouse, France.

### 2.3. Wine-Making Process

On 29 September 2024, both the disease group (DG, 16 GLRaV-3-infected plants) and control group (CK, 18 healthy plants) were harvested simultaneously. For winemaking, 20 kg of grapes were collected from each group, which were evenly divided into 3 biological replications (≈6.7 kg per replication) for separate fermentation. The grapes for each replicate were immediately destemmed and crushed. Then, 120 mg/L potassium metabisulfite was added. After 6 h, 25 mg/L pectinase EX was added. After 12 h, 200 mg/L *Saccharomyces cerevisiae* RC212 (Lallemand Inc., Montreal, QC, Canada) was inoculated to initiate alcoholic fermentation. The temperature of alcoholic fermentation was controlled at 22–26 °C. After alcoholic fermentation, the must was pressed using a manual filter press (Model Y20, Yuchang Machinery Co., Ltd., Yantai, China) to separate the wine from the pomace (skins and seeds). The obtained wine was then transferred to a separate tank for the subsequent malolactic fermentation. This addition specifies the equipment used, ensuring the process is fully documented.

Without adding SO_2_, 31MBR (Lallemand Inc., Montreal, QC, Canada) of commercial lactic acid bacteria was inoculated to start malolactic fermentation, and the temperature was maintained at 18–22 °C. After fermentation, samples were collected for analysis. During wine ageing, racking and sulfiting to 30 mg/L of free SO_2_ was performed every two months. Wine samples were taken for chemical and sensory analyses six months after fermentation ended.

### 2.4. Determination of Physicochemical Parameters

Determination of the traits of grapes. The single-berry weight (SBW) was determined using an electronic balance (FA1004, Hangping Scientific Instrument Co., Ltd., Shanghai, China), expressed as the mean of 100 fresh berries. The transverse diameter (TD) and longitudinal diameter (LD) of the grape berries were measured with an electronic vernier caliper (111-101, Anyi Instrument, Guilin, China), expressed as the mean of 30 fresh berries.

The titratable acid (TA), total sugar (TS), alcohol, volatile acid (VA), and dry extractable contents (DE) were analyzed following the methods specified in GB/T 15038-2006 “General Methods for Analysis of Wine and Fruit Wine” [17].

The pH was measured using a PHS-3C pH meter (Yidian Scientific Instrument Co., Ltd., Shanghai, China).

### 2.5. Wine Color Determination

Wine color was evaluated by the Glories parameters: color intensity (CI) and tonality (TO) [18]. The absorbance was measured at 420 nm (yellow), 520 nm (red), and 620 nm (blue) by spectrophotometer (TU-1901, Purkinje General Instrument Co., Ltd., Beijing, China).

### 2.6. Analysis of Monomeric Phenols

First, the frozen grape samples were thawed at room temperature for 3 h. Then, the stems were removed, and the samples were weighed to 100 g. Anhydrous CaCl_2_ equivalent to 1% of the weight of grapes was added, and the grapes were crushed to extract juice (without breaking the seeds). After deseeding, the juice and grape skins were transferred to a 50 mL beaker. Ultrasonic extraction (Q700, Qsonica, LLC, Newtown, CT, USA) was performed in an ice-water bath (0 °C) for 20 min. This mixture was centrifuged at 5000 r/min at 4 °C for 10 min, and the grape juice was collected for analysis [19].

#### 2.6.1. Qualitative Analysis Through UHPLC-ESI-TOF

Monomeric phenols were detected as described in Liang’s method after mild modifications [20,21]. Grape juices and wines were filtered through 0.45 μm filters and analyzed using an Agilent 1200 series HPLC system (Agilent Technologies, Santa Clara, CA, USA) equipped with a diode array detector (DAD), a binary pump, an auto-sampler, and a vacuum degasser. The HPLC system was connected to an Agilent^®^ 6520 quadrupole time-of-flight (QTOF) MS system with a dual sprayer electrospray ionization (ESI). Separation was conducted with an Agilent ZORBAX Eclipse Plus C_18_ column (4.6 × 250 mm, 5 μm) as the chromatographic column. The mobile phases included water (A) and methanol (B), and they contained 0.1% formic acid. The column temperature and injection volume were 30 °C and 10 μL, respectively. The elution was performed under the following conditions: 1 min with 5% B, 1 min with 5–16% B, 1 min with 16–20% B, 7.5 min with 20–23% B, 4 min with 23–24% B, 1 min with 24–25% B, 2.5 min with 25–26% B, 8 min with 26–27% B, 2 min with 27–28% B, 4 min with 28–30% B, 2 min with 27–28% B, 4 min with 28–30% B, 6 min with 30–35% B, 11 min with 35–45% B, 3 min with 45–47% B, 5 min with 47–48% B, 10 min with 48–50% B, 1 min with 50–80% B, 5 min with 80–100% B, 4.5 min with 100% B, and 2 min with 100–5% B at a flow rate of 1.0 mL/min. HPLC conditions were the following: column temperature 40 °C; injection volume 10 μL; and DAD detection range 190–600 nm, step 2.0 nm. Simultaneous monitoring was performed at 280, 325, and 365 nm at a flow rate of 0.5 mL/min. The MS analysis was performed in both positive and negative ionization modes with the following settings: drying gas (N_2_), nebulizer 45psi, gas flow 9 L/min, temperature 325 °C; capillary voltage 3500 V, fragmentor 175 V; and MS scan range 90–1000 *m*/*z*. The MS/MS was set in auto mode, with collision energy 15–30 eV and scan range 60–1000 *m*/*z*. Each sample was analyzed twice in positive and negative mode separately.

Data analysis was performed with MassHunter workstation software (Qualitative Analysis, version B.06.00) (Agilent Technologies, Santa Clara, CA, USA). The peak identification was conducted by comparing the retention time, UV–Vis spectra, MS, and MS/MS fragments with authentic standards and database using MassHunter workstation software and MS-DIAL 4.80 linked MS-FINDER 3.52 program. Online mass databases (ChemSpider, Phenol-Explorer, MassBank of North America) were also employed for double verification when available.

#### 2.6.2. Quantitative Analysis Through HPLC-DAD

An Agilent 1260 Infinity II Prime LC (Agilent Technologies, Santa Clara, CA, USA) connected to an Agilent 1260 series DAD was used to quantify the phenolic compounds. The column and elution condition were the same as those mentioned in qualitative analysis by HPLC-DADESI-QTOF-MS/MS. Authentic standards were employed for quantification on a linear regression of peak area against concentration. An Agilent OpenLAB workstation (Agilent Technologies, Santa Clara, CA, USA) was used for data analysis.

### 2.7. Analysis of Volatile Compounds via HS-SPME-GC-MS

The method of grape berry treatment was the same as that described in Section 2.6.

The volatile compounds were extracted [22]. The headspace solid-phase microextraction (HS-SPME) approach was used for extracting volatile compounds from grape juices and wines. First, 2 g of NaCl was weighed and placed in a 20 mL sample bottle. Next, 8 mL of grape juice or wine sample was added to the headspace bottle. Moreover, 10 μL of the internal standard 4-methyl-1-pentanol was added at 394.08 μg/L, after which the bottle was covered and sealed. The CAR/DVB/PDMS extraction fiber was inserted and allowed to adsorb for 30 min at 45 °C. The extraction fiber at the gas chromatography (GC) injection port was desorbed for 3 min at 250 °C, and then mass spectrometry (MS) analysis was conducted.

The WAX polar chromatographic column (60 m × 0.25 mm, 0.25 μm) was inverted, and the temperature was maintained at 40 °C for 5 min. Then, the temperature was increased first to 120 °C at a rate of 3 °C/min, and subsequently to 230 °C at a rate of 8 °C/min; this temperature was maintained for 10 min. The carrier gas (He) flow rate was 0.8 mL/min, and no split flow was found.

The electron impact ion source used had an energy of 70 eV, the transfer line temperature was 275 °C, the ion source temperature was 230 °C, the activation voltage was 1.5 V, the filament current was 0.25 mA, and the mass scanning range was *m*/*z* 33–450.

Using normal alkanes (C_6_–C_20_) as standards, the Kovats method was used to determine the retention indices of all compounds. Qualitative analysis was based on comparing compound retention times and RIs with those of standard compounds, a self-built library, and the NIST14 spectral library. The qualitative results were further verified with relevant published studies.

For quantitative analysis of compounds with available standards, the external standard quantitative method was used. For compounds without standard compounds, 4-methyl-1-pentanol was used as the internal standard for semiquantitative analysis [23].

### 2.8. Sensory Analysis

Ethical review and approval were waived for this study due to the use of anonymized data from routine sensory tests performed by trained staff; 11 procedures adhered to stringent ethical protocols to ensure the protection of participants’ rights. The study design incorporated the following safeguards: voluntary participation, detailed disclosure of study requirements and potential risks, and acquisition of written or verbal informed consent prior to testing. Additionally, all participant data were anonymized and securely handled to prevent unauthorized disclosure. Participants retained the right to withdraw from the study at any time without penalty. The study was conducted in full compliance with prevailing ethical standards for human subjects research.

A total of six wines (3 samples from the disease group and 3 from the control group) were tasted. The method was appropriately modified based on the descriptions of these studies [24,25]. The panel comprised 12 trained judges (5 males and 7 females) from the Changyu Longyu Estate Co., Ltd. (Yinchuan, China), all of whom were professionally trained in sensory evaluation according to ISO 8586:2023 standards [26]. Wine sensory evaluation assessed four major attributes: appearance (clarity and color), aroma (intensity, complexity, and elegance), mouthfeel (structural balance, body fullness, tannin quality, and aftertaste), and overall quality (Cabernet Sauvignon typicity). Panelists rated the intensity of each parameter using a scale (0 = absence, 1–3 = low, 4–6 = middle, 7–9 = high, and 10 = max). To ensure evaluation consistency, each panelist assessed the same sample at least twice.

Wine samples, labeled with random three-digit codes, were presented in ISO tasting glasses at room temperature (20 °C) in a specialized tasting room. The wine serving temperature was 16 °C, with 40 mL poured into each glass for simultaneous tasting. Tasting was conducted from 10 to 11 a.m.

### 2.9. Statistical Analysis

All data were expressed as mean ± standard deviation from triplicates. Statistical analyses of each indexes between control group and disease group were performed using GraphPad Prism 8 software (GraphPad Software, LLC, San Diego, CA, USA). A two-way ANOVA was applied to evaluate significance, with *p* < 0.01 considered statistically significant and *p* < 0.001 considered highly significant. R 4.0.4 (RStudio, Boston, MA, USA) was used to make a heatmap for analysis of the content differences of monomeric phenols and a Radar chart for analysis of the difference of sensory scores for wines. Origin Pro 2018 (OriginLab, Northampton, MA, USA) was used to produce a clustered column chart of physicochemical parameters, the polyphenolic and volatile organic compounds. Principal component analysis (PCA) of the polyphenolic and volatile organic compounds was performed using the SIMCA-P 15.0 (Sartorius, Umeå, Sweden).

## 3. Results and Discussion

### 3.1. Effects of GLRaV-3 on the Physicochemical Parameters of Cabernet Sauvignon Grapes and Wines

**Figure 1:**
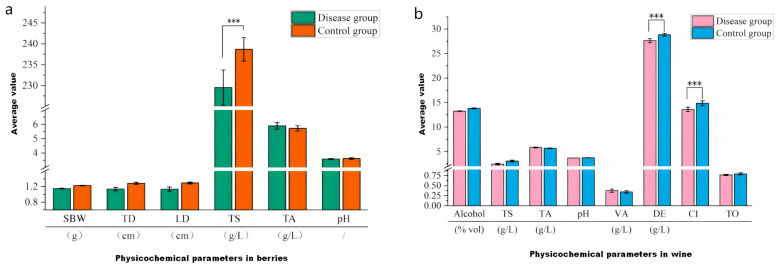
Effect of GLRaV-3 on the characteristics and physicochemical parameters of Cabernet Sauvignon berries (**a**) and wines (**b**). Note: The GraphPad Prism 8 software was used to conduct two-way ANOVA (***: highly significant (*p* < 0.001)), and the same is true for other graphs (SBW: single-berry weight, TD: transverse diameter, LD: longitudinal diameter, TS: total sugar, TA: titratable acid, VA: volatile acid, DE: dry extractable contents, CI: color intensity, and TO: tonality).

Comparisons of the main traits and physicochemical parameters of the grape berries between the GLRaV-3 disease group and the control group are shown in Figure 1a and Table S1.The results revealed that GLRaV-3 infection decreased the mass and size of Cabernet Sauvignon berries compared to the control group, as indicated by lower single-berry weight (SBW), transverse diameter (TD), and longitudinal diameter (LD). Regarding total sugar content, the control group had a significantly greater average value of 238.66 g/L than the disease group (229.53 g/L) (*p* < 0.001), indicating that GLRaV-3 negatively affects sugar accumulation. This finding was similar to those of studies by Akbaş et al. [13] and Han et al. [11]. The observed effects may be attributed to alterations in sugar transporter genes and transcript levels related to sugar metabolism caused by GLRaV-3 [7]. Leafroll disease suppresses photosynthesis, thus negatively affecting sugar accumulation [27]. GLRaV-3, a phloem-limited filamentous virus, induces hyperplasia in the vascular bundle parenchyma and phloem tissues of leaf veins, disrupting sugar accumulation and transport [14]. In contrast, the total acid content and pH did not differ significantly between the groups.

A comparison of the physicochemical parameters of wines produced from GLRaV-3-infected grapes and healthy control grapes is shown in Figure 1b and Table S2. Our findings revealed that dry extract and color intensity (CI) were highly significantly different (*p* < 0.001) between the groups. The disease group presented significantly lower values of dry extract (27.62 g/L) and CI (13.60) than the control group (28.83 g/L and 14.86, respectively). These findings suggest that grapevine leafroll disease (associated with GLRaV-3) may negatively affect the sensory profile (e.g., taste) and color characteristics of wine. Additionally, the alcohol and total sugar content of wines from the disease group decreased relative to the control group, matching the decreased sugar levels observed in the infected grape berries. Numerous studies confirm that GLRaV-3 infection significantly delays grapevine phenological development during the growing season and causes a post-veraison ripening delay [10,11].

Compared to those in the control group, the CI in wines from the disease group was significantly lower (*p* < 0.001). Color intensity, a critical sensory attribute of wine, is determined primarily by pigments such as anthocyanins. GLRaV-3 disrupts photosynthesis and metabolic processes in grape leaves [7,27], thereby impairing anthocyanin synthesis and accumulation. This aligns with Han et al. [11], who reported reduced anthocyanin content in GLRaV-3-infected Cabernet Sauvignon berries due to viral interference with leaf photosynthetic capacity.

### 3.2. Effect of GLRaV-3 on Monomeric Phenols in Cabernet Sauvignon Grapes and Wines

**Figure 2:**
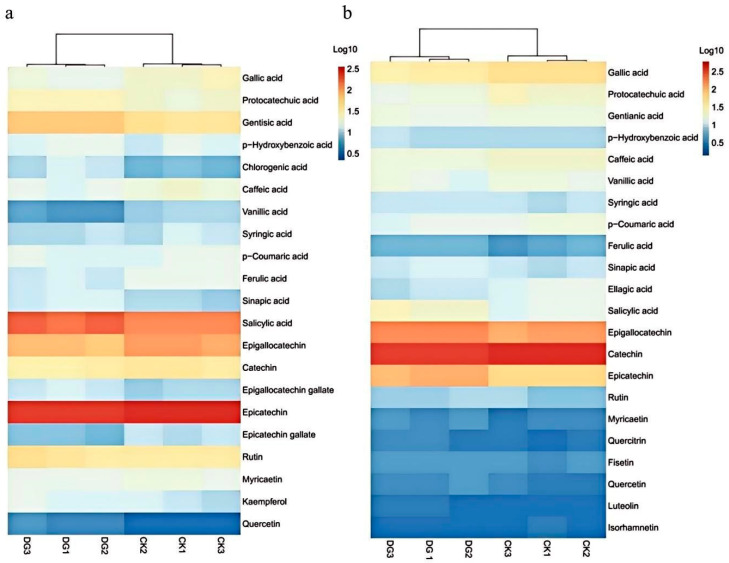
Heatmap of monomeric phenols in Cabernet Sauvignon berries (**a**) and wines (**b**) (DG: disease group, CK: control group).

Monomeric phenols were detected in the diseased grape group and the control group, and the results are shown in Figure 2 and Tables S3–S6. A total of 21 monomeric phenols were detected in grape berries, including twelve phenolic acids, five flavanols, and four flavonols (Figure 2a). A total of 22 monomeric phenols were detected in wine, including twelve phenolic acids, three flavanols, and seven flavonols (Figure 2b).

#### 3.2.1. Phenolic Acids

Phenolic acids are important group of small-molecule compounds in wine; they primarily include benzoic acid, cinnamic acid, and their derivatives [28]. These compounds play key roles in determining wine color stability, flavor complexity, aroma profile, and antioxidant properties [29].

Comparison of phenolic acids in grape berries and wines revealed that the total content of phenolic acids was 382.77 mg/L and 312.57 mg/L in GLRaV-3-infected and healthy grape berries, respectively, while the corresponding contents in wines decreased to 164.93 mg/L and 195.27 mg/L. Among these, the contents of salicylic acid and gentisic acid dropped significantly. This phenomenon may be jointly influenced by multiple factors, including extraction rate, enzymatic and microbial oxidation, clarifier adsorption, and polymerization [29,30,31]. For example, Lisov et al. fermented Cabernet Sauvignon grapes using different winemaking techniques and found that the extraction rates of various phenolic acids varied with the use of different enzyme preparations and maceration times [31].

**Figure 3:**
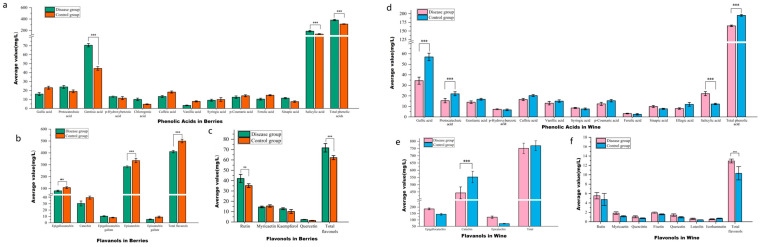
Effect of GLRaVs on phenolic acids, flavanols, and flavonoids in Cabernet Sauvignon berries (**a**–**c**) and wines (**d**–**f**). Note: The GraphPad Prism 8 software was used to conduct two-way ANOVA (***: highly significant (*p* < 0.001); ** : significant (*p* < 0.01)).

A comparative analysis of 12 phenolic acids in grape berries between GLRaV-3-infected and healthy Cabernet Sauvignon is shown in Figure 3a and Table S3.

The content of total phenolic acid in the disease group was significantly greater (382.77 mg/L) than that in the control group (312.57 mg/L, *p* < 0.001). The contents of five phenolic acids increased, while those of seven phenolic acids decreased. After grapes are infected by fungi or viruses, changes in phenolic compounds often show a pattern of decreased specific monomers but increased total phenolic content. The combined effects of cell wall destruction caused by infection, phenolic oxidation/polymerization, enhanced synthesis of defensive phenolics in plants, and differences in extraction methods lead to reduced concentrations of specific phenolic monomers. In contrast, the total phenolic content increases due to the release and polymerization of phenolics and the production of new phenolic compounds [32,33]. The contents of glyceric acid and salicylic acid, which are the two predominant phenolic acids in Cabernet Sauvignon berries, were extremely significantly different (*p* < 0.001). The disease group presented significantly greater percentages of gentisic and salicylic acids (70.58 and 188.40 mg/L, respectively) than the control group (44.64 and 136.83 mg/L, respectively). Studies suggest that GLRaV-3 infection activates the plant immune system, triggering defense responses. Specifically, it induces salicylic acid-associated signaling pathways, leading to greater salicylic acid accumulation [34]. Additionally, viral infection suppresses photosynthesis, leading to the accumulation of carbohydrates and organic acids in leaves. These accumulated organic acids are metabolized into phenolic acids via biosynthetic pathways [35].

A comparison of the 12 phenolic acids in wines produced from grapes in the GLRaV-3-infected group and the healthy control group is shown in Figure 3d and Table S4. The contents of gallic acid, salicylic acid, protocatechuic acid, and total phenolic acids were extremely significantly different (*p* < 0.001) between the groups. The control group presented significantly greater levels of gallic acid (56.99 mg/L), protocatechuic acid (21.98 mg/L), and total phenolic acids (195.27 mg/L) than the infected group (34.44, 15.45, and 164.93 mg/L, respectively). However, the salicylic acid content in the infected group (22.08 mg/L) was significantly higher than in the control group (11.28 mg/L), matching the grape analysis results. The total amount of phenolic acids in wine made from leafroll virus-infected grapes was lower than in the control group, with significantly lower gallic and protocatechuic acid contents. Viral infection may change the thickness of the fruit skin and the cellular structure, thus affecting the amount of phenolic compounds extracted. In Nebbiolo grapes, mixed infections (GFLV, GFkV, GLRaV-3, and GVA) induced changes in berry skin mechanical properties, most evident in skin thickening, which decreases phenol extractability from infected vines [36].

#### 3.2.2. Flavanols

Flavanols are important compounds that contribute to the bitterness and astringency of wine, and they mainly originate from the skins and seeds of grapes [37]. Comparison of flavanols between grape berries and their corresponding wines showed that the total content of flavanols in wines was higher than that in grape berries, being 1.82 times higher in the control group and 1.50 times higher in the GLRaV-3 infected group, respectively. EGCG and ECG were only detected in grape berries. This is mainly because enzymatic hydrolysis during wine fermentation breaks ester bonds, producing gallic acid and the corresponding non-esterified catechins (EGC and EC) [38].

A comparison of the flavanols in wines made from grape berries infected with GLRaV-3 with those made from healthy control grape berries is shown in Figure 3b,e and Tables S5 and S6. The results showed that in grape berries, epigallocatechin and epicatechin are the main flavanol compounds. The average values in the control group (107.96 mg/L and 334.83 mg/L) were significantly higher than those in the infected group (80.67 mg/L and 283.58 mg/L) (*p* < 0.01), and correspondingly, the total flavanol content of the control group was significantly higher than that of the infected group (*p* < 0.001). The contents of catechin and epigallocatechin gallate (EGCG) in the control group were higher than those in the infected group, but the difference was not significant. In the wines produced, the content of catechin (CAT) in the control group (553.39 mg/L) was higher than that in the infected group (443.44 mg/L) (*p* < 0.001), and the total amount of flavanols was also higher in the control group. While the contents of epigallocatechin (EGC) and epicatechin (EC) were greater in the infected group than in the control group, the differences between the groups were not significant.

In summary, GLRaV-3 infection decreases flavanol compounds in both Cabernet Sauvignon berries and wines. This occurs mainly because the synthesis of flavanol compounds requires sufficient photosynthetic products as precursor compounds. Infection by grape viruses decreases the photosynthetic efficiency of grape leaves, which in turn affects the accumulation of phenolic compounds in berries, leading to a decrease in flavanol content [39].

#### 3.2.3. Flavonols

Flavonols are derivatives of flavanols. In Cabernet Sauvignon grapes and wines, the main flavonols include rutin, quercetin, and myricetin. These flavonols contribute to wine color formation and significantly affect antioxidant properties and sensory characteristics [28]. Comparison of grape berries and their corresponding wines revealed that the total flavonol content in the control and GLRaV-3 infected grape berries was 71.72 mg/L and 62.22 mg/L, respectively, which was significantly higher than that in the corresponding wines (12.94 mg/L and 10.32 mg/L). However, two flavonol compounds (luteolin and isorhamnetin) were detected only in the wines.

A comparison of the flavonols in wines made from grape berries infected with GLRaV-3 and those from healthy control grape berries is shown in Figure 3c,f and Tables S5 and S6. The results revealed that in grape berries, the average value of rutin in the infected group (42.05 mg/L) was significantly higher than that in the control group (35.18 mg/L) (*p* < 0.01). Moreover, the total flavonol level in the infected group (71.72 mg/L) was also significantly higher than that in the control group (62.22 mg/L) (*p* < 0.001). This may affect aspects such as grape antioxidant activity. In the wines produced, the total amount of flavonols in the infected group (12.94 mg/L) was significantly higher than that in the control group (10.32 mg/L) (*p* < 0.001), whereas the contents of other individual flavonol compounds did not differ significantly between the groups. In summary, GLRaV-3 infection significantly increased flavonol content in both Cabernet Sauvignon berries and their wines. Viral infection may change the response of grape plants to environmental stress, thus affecting the accumulation of flavonols [40]. Viral infection may also activate certain genes related to flavonol synthesis, thus increasing flavonol content, which needs to be verified by subsequent studies.

Overall, GLRaV-3 can influence specific phenolic compound compositions and levels in wine, which may, in turn, have potential effects on the quality characteristics of wine, such as its flavor, color, and antioxidant properties.

#### 3.2.4. Principal Component Analysis (PCA) of Phenolic Compounds

**Figure 4:**
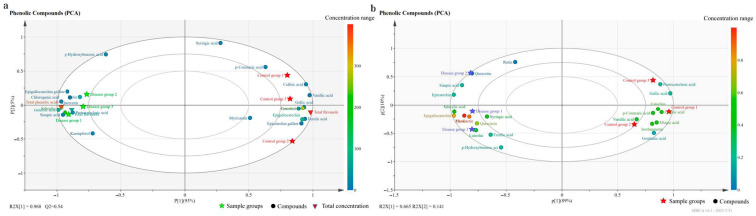
PCA of phenolic compounds in Cabernet Sauvignon berries (**a**) and wines (**b**).

Figure 4a shows the principal component analysis (PCA) results of phenolic compounds in grape berries between the GLRaV-3-infected group and the control group. The first principal component (P1) explained 93% of the variance, with R2X[1] = 0.968, indicating a good model fit. The infected and control groups were well-separated in the principal component space. The samples of the infected group were distributed in the second and third quadrants, showing a negative correlation with P1, whereas the samples of the control group were distributed in the first and fourth quadrants, showing a positive correlation with P1. These findings indicated that the monomeric phenolic compound composition significantly differed between the two groups. Regarding specific compound distribution, 11 indices (e.g., caffeic acid, gallic acid, ferulic acid, vanillic acid, EGC, EC, epicatechin gallate, and total flavanols) were relatively concentrated around the control group sample area, indicating higher contents in the control group. In contrast, 12 indices (including gentisic acid, salicylic acid, chlorogenic acid, sinapic acid, total phenolic acids, and EGCG) were mostly distributed around the disease group sample area, indicating greater contents in the disease group.

The PCA results of the phenolic compound indices of the wines made from the grapes of the disease group with GLRaV-3 and the uninfected control group are shown in Figure 4b. P1 from PCA explained 89% of the variance. The results showed that the two groups of samples were well-differentiated in the PCA diagram. The samples of the infected group were distributed in the second and third quadrants, and they were negatively related to P1, whereas the samples of the control group were distributed in the first and fourth quadrants and were positively correlated with P1. These findings indicate significant differences in monomeric phenolic compound composition between the two groups. Eight monomeric phenols, such as gallic acid, caffeic acid, protocatechuic acid, and CAT, were distributed on the side of the control group, and all were within the confidence ellipse interval, indicating that these monomeric phenols were strongly associated with the control group. While 13 monomeric phenols, such as salicylic acid, EGC, EC, myricetin, and quercetin, were relatively concentrated on the side of the disease group and all within the confidence ellipse interval, these monomeric phenols were strongly associated with the disease group.

The PCA results also revealed that GLRaV-3 significantly affects the phenolic compound composition and content distribution in grape berries and wines. Consequently, it may have different effects on the quality and characteristics of wine, such as its flavor and antioxidant properties.

### 3.3. Effects of GLRaV-3 on Volatile Compounds in Cabernet Sauvignon Grapes and Wines

#### 3.3.1. Differences in Volatile Compound Contents

Volatile compounds are important flavor compounds in wine grapes and wines. Their types, contents, sensory thresholds, and interactions determine grape flavor quality, as well as the flavor and typicality of the resulting wines [41,42]. In total, 46 volatile compounds were detected in Cabernet Sauvignon grape berries (Table 1), which can be classified into four esters, seven alcohols, three fatty acids, eight aldehydes, ten terpenes, eight C_6_ compounds, and six aromatic compounds. Among them, the total proportion of aromatic compounds, alcohols, terpenes, and esters was over 95%.

**Figure 5:**
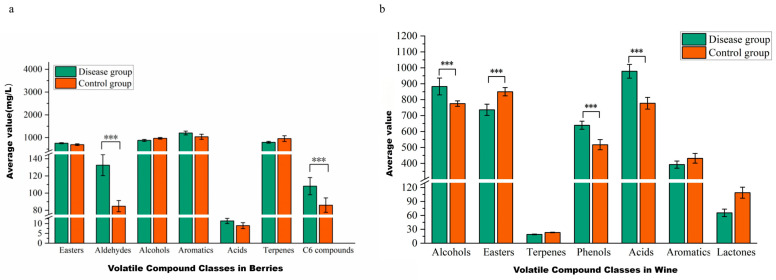
Effect of GLRaVs-3 on volatile compounds in Cabernet Sauvignon berries (**a**) and wines (**b**). Note: The GraphPad Prism 8 software was used to conduct two-way ANOVA (***: highly significant (*p* < 0.001).

A comparison of the volatile compounds in grape berries in the GLRaV-3-infected and control groups is shown in Figure 5a. In the disease group, the contents of esters, aldehydes, aromatic compounds, and C_6_ compounds were higher than in the control group, whereas alcohols and terpenes were lower. Significant differences were found in the contents of aldehydes and C_6_ compounds (*p* < 0.001), and their contents (132.47 mg/L and 108.40 mg/L) in the disease group were greater than those (84.77 mg/L and 85.95mg/L) in the control group. The increase in aldehydes was related mainly to the significantly greater content of decanal in the infected group. The increase in C_6_ compounds was mostly associated with a significant increase in the content of (E)-2-hexen-1-ol in the infected group. This is attributed to GLRaV-3-mediated interference in sugar metabolism during fruit ripening, as supported by Akbaş et al. [13] who reported viral disruption of carbohydrate metabolism in infected grapevines. Consistent with previous observations, C_6_ aldehyde concentrations increase in GLRaV-3-infected berries during early ripening and decline as ripening proceeds [12].

In total, 57 aroma compounds, which can be classified into fifteen esters, thirteen alcohols, nine fatty acids, five terpenes, seven volatile phenols, seven aromatic compounds, and one lactone, were detected in Cabernet Sauvignon wines (Table 2). Among them, fatty acids, esters, and alcohols are major volatile compounds. Compared with grape berries, the variety and content of aroma compounds in wines increased significantly, mainly due to the massive production of esters, alcohols, fatty acids and other substances during the fermentation [42,43].

A comparison of volatile compounds in the wines of the group infected with GLRaV-3 and the control group is shown in Figure 5b. The results showed significant differences in alcohols, phenols, fatty acids, and esters between the infected group and the control group. The alcohol, phenol, and fatty acid contents (882.70, 639.21, and 978.19 mg/L, respectively) were greater than those in the control group (775.53, 517.07, and 777.51 mg/L, respectively), whereas the content of esters (736.39 mg/L) was lower than that in the control group (849.91 mg/L). Esters can impart wine with fruity and floral aromas. Greater grape berry ripeness and higher sugar content generally lead to greater ester content [44], similar to findings for Cabernet Gernischt grapes in the eastern foot of the Ningxia Helan Mountains [12].

#### 3.3.2. The PCA of Volatile Compounds

**Figure 6:**
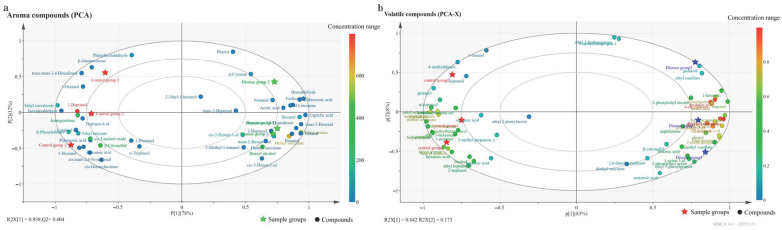
PCA of aroma compound indices in Cabernet Sauvignon berries (**a**) and wines (**b**).

The PCA results of the disease group with GLRaV-3 and the control group based on 46 aroma compound indices of the grape berries are shown in Figure 6a. The first principal component P1 explained 78% of the variance, and the second principal component P2 explained 12% of the variance. The R2X[1] value (0.938) indicated good model fit. The results revealed prominent differences in the principal component space between the disease and control groups. The disease group was distributed mainly in the first and fourth quadrants, showing a positive correlation with P1, whereas the control group was distributed mainly in the second and third quadrants, showing a negative correlation with P1, indicating that the aroma compound composition significantly differed between the two groups of samples. Among them, eight compounds, such as ethyl isovalerate, isovaleraldehyde, β-phenylethyl alcohol, linalool, 1-heptanol, terpinolene, ethyl butyrate, and 1-hexanol, were concentrated around the area of the control group (P1 ≤ −0.80), indicating that the contents of these aroma compounds were higher in the control group. In total, 12 compounds, including caprylic acid, benzaldehyde, (E)-2-hexenal, ethyl acetate, caprylaldehyde, decanal, 2-hexenoic acid, (E,E)-2,4-heptadienal, furfural, hexanal, D-limonene, and methyl salicylate, were distributed near the area of the disease group (P1 ≥ 0.80), indicating that the contents of these aroma compounds were higher in the disease group.

The PCA results of the aroma compounds in the wines of grape berries from the GLRaV-3-infected group and the control group are shown in Figure 6b. P1 explained 83% of the variance, and P2 explained 8% of the variance. Considerable differences were observed in the principal component space between the disease and control groups, indicating significant differences in aroma compound composition. The control group was found in the second and third quadrants. In total, 12 volatile compounds, including ethyl nonanoate, isoamyl acetate, benzaldehyde, ethyl caprylate, 1-pentanol, butyrolactone, geraniol, ethyl caprate, methyl caprylate, terpineol, hexyl acetate, benzyl alcohol, and caproic acid (P1 ≤ −0.80), were distributed on the side of the control group, indicating that the contents of these compounds were higher in the control group. The disease group was located in the first and fourth quadrants. In total, 12 volatile compounds, including eugenol, palmitic acid, ethyl palmitate, 1-hexanol, 2-ethyl-1-hexanol, (Z)-2-hexen-1-ol, 2-methylpropanol, ethyl hexanoate, 4-vinylguaiacol, ethyl cinnamate, capric acid, and ethyl vanillate (P1 ≥ 0.90), were distributed on the side of the disease group, indicating that these compounds are more closely related to the disease group.

To summarize, GLRaV-3 significantly affects the composition and content distribution of volatile compounds in grape berries and wines, which leads to changes in the aroma characteristics of wines.

### 3.4. Sensory Evaluation Analysis of Cabernet Sauvignon Wines

**Figure 7:**
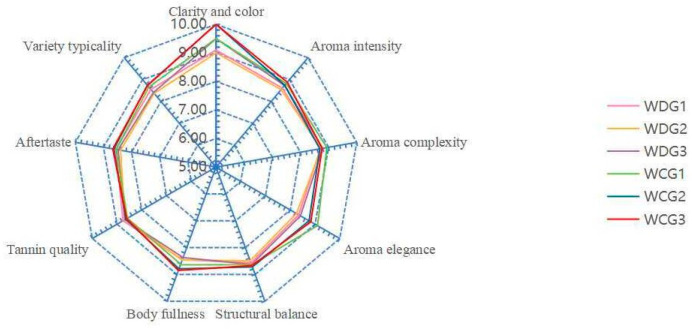
Radar chart of sensory scores for Cabernet Sauvignon wines (WDG: disease group, WCG: control group).

A comprehensive sensory analysis was performed on wines from GLRaV-3-infected (disease group) and healthy grapes (control group), evaluating four principal attributes: appearance, aroma, mouthfeel, and overall quality (Figure 7 and Table S7). The sensory outcomes align closely with the physicochemical and chemical data, confirming that GLRaV-3 infection systematically compromises wine quality.

Visually, wines from the disease group displayed significantly lighter color than those from the control group (mean scores: 9.20 vs. 9.83). This observation is consistent with the reduced color intensity (CI) measured instrumentally. The lower anthocyanin synthesis in infected grapes likely explains this diminished color depth, adversely affecting the visual typicity of the wine.

Regarding aroma, wines were assessed for intensity, complexity, and elegance. The disease group scored lower in both intensity and elegance (mean scores: 8.63 and 8.33) compared to the control (8.81 and 8.90), while complexity scores were comparable. Notably, wines from infected grapes exhibited distinct green and pungent aromatic notes. This sensory profile corresponds to higher concentrations of C_6_ compounds—such as (E)-2-hexen-1-ol, 1-hexanol, (E)-2-hexenal, and hexanal—along with elevated levels of 2-methylpropanol, compounds typically associated with vegetative and herbaceous odors. In contrast, control wines were characterized by more pronounced fruity and sweet notes, attributable to their higher ester content, including isoamyl acetate, ethyl octanoate, and ethyl decanoate, which are key contributors to floral and fruity aromas.

Regarding mouthfeel, the disease group received lower ratings for structural balance and body fullness (mean scores: 8.56 and 8.43) than the control (8.68 and 8.75). These results correlate with the lower alcohol, dry extract, phenolic acids, and sugar content measured in wines from infected grapes, resulting in a lighter and less substantial mouthfeel. Conversely, the disease group outperformed the control in tannin quality and aftertaste persistence (mean scores: 8.81 and 8.74 vs. 8.61 and 8.52). This improvement may be linked to shifts in phenolic composition: although total flavanol content was reduced in the disease group, the relative increase in flavonols and certain monomeric phenolic acids may have contributed to a smoother and better-integrated tannin structure. Meanwhile, control wines, with higher total phenols and lower acidity, presented a more astringent and heavy tannin sensation, which likely diminished their perceived tannin finesse and aftertaste clarity.

Finally, the overall quality score was lower for wines from the infected group (8.41) than for the control (8.74). This indicates that GLRaV-3 infection not only alters specific sensory attributes but also impairs the overall typicity and stylistic consistency of Cabernet Sauvignon wines. The decline in overall quality results from the combined effects of reduced color intensity, shifted aroma profile, weakened body structure, and modified mouthfeel balance, collectively leading to a deviation from the expected varietal style.

In conclusion, GLRaV-3 infection modifies grape chemical composition, which in turn systematically influences the sensory attributes of the resulting wine. The strong agreement between sensory evaluation and analytical data provides robust organoleptic evidence for the impact of viral infection on wine quality. These findings offer clear direction for potential adjustments in winemaking practices and quality management strategies in affected vineyards.

## 4. Conclusions

This study reveals that GLRaV-3 infection systematically modifies the composition and sensory attributes of grapes and wine. Infected grapes showed reduced sugar content (4.8% decrease) and altered acid levels, resulting in wines with lower alcohol levels and reduced dry extract. These changes weakened wine body and varietal intensity. Phenolic metabolism responded differently in grapes and wine. Grape berries exhibited increased phenolic acids (22.46% higher) and flavonols (15.27% higher), but decreased flavanols (17.86% lower). After fermentation, however, phenolic acids decreased by 18.40%, while flavonols remained elevated (12.54% higher). Volatile profiles were also altered. Aldehydes (56.27% higher) and C_6_ compounds (26.12% higher) increased in grapes, whereas wines displayed more alcohols and phenols but fewer esters (13.36% lower). Sensorially, these changes improved tannin texture and aftertaste but reduced color depth, aromatic complexity, and varietal typicity.

These findings provide a basis for adapting viticultural and winemaking practices in affected regions. However, how GLRaV-3 regulates metabolic pathways remains unclear. Future studies should examine its effects on key enzymes and signaling during grape ripening.

## Figures and Tables

**Table 1:** The volatiles in Cabernet Sauvignon berries from the disease group and the control group (mg/L).

RI	Indexes	Grapes from the Disease Group				Grapes from the Control Group			
		Sample 1	Sample 2	Sample 3	Average (SD)	Sample 1	Sample 2	Sample 3	Average (SD)
	**Esters**								
890	Ethyl acetate	605.22	581.63	628.81	605.22 ± 23.59 b	482.03	508.84	458.28	483.05 ± 25.30 a
1031	Ethyl butyrate	77.92	69.73	82.11	76.59 ± 6.30 a	87.49	92.9	85.47	88.62 ± 3.84 b
1045	Ethyl isovalerate	70.37	80.45	64.29	71.70 ± 8.16 a	120.84	122.74	112.28	118.62 ± 5.572 b
1695	Diethyl succinate	1.03	0.73	0.91	0.89 ± 0.15 b	0.5	0.87	0.43	0.60 ± 0.24 a
	**Aldehydes**								
921	Isovaleraldehyde	ND	ND	ND	ND	0.22	0.33	0.20	0.25 ± 0.07 b
1293	Octanal	6.12	4.96	5.74	5.61 ± 0.59 b	3.18	3.69	2.97	3.28 ± 0.37 a
1345	(E)-2-Heptenal	3.19	3.95	2.23	3.12 ± 0.86 a	2.87	2.67	2.02	2.52 ± 0.44 a
1428	Nonanal	19.66	22.85	17.47	19.99 ± 2.71 b	16.49	17.40	15.37	16.42 ± 1.02 a
1453	(E,E)-2,4-Heptadienal	5.35	4.62	6.12	5.36 ± 0.75 b	3.46	2.80	3.63	3.30 ± 0.44 a
1475	Furfural	7.17	8.43	6.91	7.50 ± 081 b	5.35	3.63	5.19	4.72 ± 0.95 a
1480	Decanal	89.77	97.31	79.23	88.77 ± 9.08 b	54.94	55.23	45.65	51.94 ± 5.45 a
1569	(Z,E)-2,6-Nonadienal	2.27	1.99	2.08	2.11 ± 0.14 a	2.53	2.25	2.24	2.34 ± 0.16 a
	**Alcohols**								
1116	(Z)-2-Penten-1-ol	114.76	83.86	119.62	106.08 ± 19.40 a	91.32	86.16	103.38	93.62 ± 8.84 a
1214	3-Methylbutanol	0.19	0.13	0.15	0.16 ± 0.03 a	0.12	0.16	0.11	0.13 ± 0.03 a
1251	1-Pentanol	7.73	7.06	7.4	7.40 ± 0.34 a	7.51	8.4	7.28	7.73 ± 0.59 a
1318	2-Heptanol	2.25	1.79	1.71	1.92 ± 0.29 b	1.04	1.81	0.91	1.25 ± 0.49 a
1458	1-Heptanol	709.37	749.62	787	748.66 ± 38.82 a	831.12	899.87	817.87	849.62 ± 44.02 b
1483	2-Ethyl-1-hexanol	2.79	2.45	2.58	2.61 ± 0.17 a	2.28	2.75	2.71	2.58 ± 0.26 a
1573	1-Octanol	11.42	12.54	10.48	11.48 ± 1.03 a	15.02	12.54	15.88	14.48 ± 1.73 b
	**Aromatic compounds**								
1570	Benzaldehyde	8.08	9.72	8.9	8.90 ± 0.82 b	5.34	5.55	6.18	5.69 ± 0.44 a
1667	Phenylacetaldehyde	25.56	37.98	31.77	31.77 ± 6.21 a	32.76	35.44	39.17	35.79 ± 3.22 a
1751	Methyl salicylate	729.21	645.45	687.33	687.33 ± 41.88 b	590.22	465.92	539.57	531.90 ± 62.50 a
1860	Benzyl alcohol	396.55	314.35	355.45	355.45 ± 41.10 b	331.43	262.41	306.82	300.22 ± 34.98 a
1898	β-Phenylethanol	113.57	99.44	127.7	113.57 ± 14.13 a	178.54	144.12	155.15	159.27 ± 17.58 b
1997	Phenol	4.54	5.68	5.11	5.11 ± 0.57 a	2.85	4.30	6.19	4.45 ± 1.68 a
	**Acids**								
1435	Acetic acid	1.79	3.03	2.66	2.49 ± 0.64 b	1.62	1.36	1.22	1.40 ± 0.20 a
2057	Caprylic acid	3.27	4.04	3.92	3.74 ± 0.41 b	1.24	0.93	1.06	1.08 ± 0.16 a
2165	Nonanoic acid	4.79	4.75	5.77	5.10 ± 0.58 a	7.83	5.55	6.09	6.49 ± 1.19 b
	**Terpenes**								
1190	d-Limonene	5.53	4.77	6.24	5.51 ± 0.74 b	2.47	3.91	4.35	3.58 ± 0.98 a
1331	Isoterpinolene	251.10	261.43	232.77	248.43 ± 14.51 a	288.15	302.36	263.94	284.81 ± 19.43 b
1468	(Z)-Linalool oxide	212.58	186.91	248.25	215.91 ± 30.81 a	271.54	317.25	235.83	274.87 ± 40.81 b
1551	Linalool	0.22	0.10	0.18	0.17 ± 0.06 a	0.27	0.31	0.27	0.28 ± 0.02 b
1584	Terpinen-4-ol	2.76	2.58	1.94	2.43 ± 0.43 a	5.30	2.85	4.24	4.13 ± 1.23 b
1683	α-Terpineol	3.37	3.65	4.19	3.74 ± 0.42 a	4.74	3.52	3.71	3.99 ± 0.66 a
1779	β-Citronellal	306.35	238.79	262.57	269.24 ± 34.27 a	342.07	407.37	266.77	338.74 ± 70.36 b
1807	β-Damascenone	4.73	5.40	3.49	4.54 ± 0.97 a	5.62	6.23	7.84	6.56 ± 1.15 b
1876	(Z)-Geranylacetone	0.78	0.59	0.77	0.71 ± 0.11 a	1.44	0.86	0.74	1.01 ± 0.37 b
2176	p-Cymene	37.25	42.92	46.59	42.25 ± 4.71 b	31.89	39.29	42.47	37.88 ± 5.43 a
	**C_6_ compounds**								
1073	Hexanal	3.25	1.69	2.67	2.54 ± 0.79 b	0.81	0.93	1.09	0.94 ± 0.14 a
1192	(E)-2-Hexenal	4.91	4.24	5.64	4.93 ± 0.70 b	2.58	2.60	3.00	2.73 ± 0.24 a
1348	1-Hexanol	0.72	0.69	0.85	0.75 ± 0.09 a	1.14	0.97	0.84	0.98 ± 0.15 b
1377	(Z)-3-Hexen-1-ol	6.16	6.18	7.20	6.51 ± 0.59 a	6.19	4.47	2.85	4.50 ± 1.67 a
1395	(E,E)-2,4-Hexadienal	0.69	0.75	0.56	0.67 ± 0.10 a	0.84	0.95	1.12	0.97 ± 0.14 b
1399	(E)-2-Hexen-1-ol	79.90	89.71	99.12	89.58 ± 9.61 b	80.28	73.82	66.36	73.48 ± 6.97 a
1843	Hexanoic acid	1.16	1.10	1.08	1.11 ± 0.04 a	1.57	1.28	1.19	1.35 ± 0.20 b
1960	2-Hexenoic acid	2.01	2.23	1.61	1.95 ± 0.31 b	0.93	0.89	1.15	0.99 ± 0.14 a
	**Totals**	3947.41	3712.27	3979.17	3879.62 ± 145.79 a	3927.97	3920.48	3651.082	3833.18 ± 157.74 a

Different letters (a, b) indicate significant differences according to the Tukey HSD test (*p* < 0.05).

**Table 2:** The volatiles in Cabernet Sauvignon wines of the disease group and the control group (mg/L).

RI	Indexes	Grapes from the Disease Group				Grapes from the Control Group			
		Sample 1	Sample 2	Sample 3	Average (SD)	Sample 1	Sample 2	Sample 3	Average (SD)
	**Alcohols**								
1110	2-Methylpropanol	273.67	258.15	299.19	277.00 ± 20.72 b	201.93	194.7	219.16	205.26 ± 12.57 a
1251	1-Pentanol	107.3	85.68	98.92	97.30 ± 10.90 a	137.73	139.36	156.1	144.40 ± 10.17 b
1318	2-Heptanol	5.18	4.36	5.6	5.05 ± 0.63 a	6.47	5.36	7.08	6.30 ± 0.87 a
1348	1-Hexanol	6.61	7.14	5.28	6.34 ± 0.96 b	2.33	2.94	2.42	2.56 ± 0.33 a
1377	(Z)-3-hexen-1-ol	298.29	285.09	331.49	304.96 ± 23.91 b	250.48	270.71	240.25	253.81 ± 15.50 a
1399	(E)-2-hexen-1-ol	129.69	113.59	120.79	121.36 ± 8.06 b	81.01	80.92	91.1	84.34 ± 5.85 a
1289	1-Octen-3-ol	16.29	13.92	15.66	15.29 ± 1.23 a	13.2	12.9	14.4	13.50 ± 0.79 a
1458	1-Heptanol	21.43	23.19	21.87	22.16 ± 0.92 a	29.49	33.77	23.21	28.82 ± 5.31 b
1483	2-Ehyl-1-hexanol	1.27	1.72	1.82	1.60 ± 0.29 b	0.45	0.38	0.72	0.52 ± 0.18 a
1512	2-Nonanol	2.62	3.04	3.2	2.95 ± 0.30 b	2.28	1.76	2.17	2.07 ± 0.27 a
1573	1-Octanol	14.29	16.56	13.02	14.62 ± 1.79 a	16.33	19.51	15.15	17.00 ± 2.26 a
1669	1-Nonanol	11.42	12.07	13.77	12.42 ± 1.21 a	13.78	15.39	17.17	15.45 ± 1.70 a
1749	3-Methylthiopropan-1-ol	1.69	1.97	1.27	1.64 ± 0.35 a	1.41	1.75	1.32	1.49 ± 0.23 a
	**Esters**								
1125	3-Methylbutanol acetate	1.02	0.85	1.49	1.12 ± 0.33 a	3.07	3.36	3.88	3.44 ± 0.41 b
1241	Ethyl hexanate	1.39	1.01	1.59	1.33 ± 0.29 b	0.46	0.49	0.53	0.49 ± 0.04 a
1258	Hexyl acetate	0.52	0.51	0.59	0.54 ± 0.04 a	0.69	0.63	0.75	0.69 ± 0.06 b
1290	Ethyl 3-hexenoate	1.03	0.92	1.24	1.06 ± 0.16 a	1.32	1.44	1.26	1.34 ± 0.09 b
1352	Ethyl 2-hydroxypropanoate	71.03	79.83	66.23	72.36 ± 6.90 a	66.08	76.73	65.43	69.41 ± 6.34 a
1370	Ethyl heptanoate	1.13	1.09	1.31	1.18 ± 0.12 a	1.33	1.26	1.59	1.39 ± 0.17 a
1410	Methyl octanoate	11.11	8.69	9.53	9.78 ± 1.23 a	12.83	13.95	12.71	13.16 ± 0.68 b
1450	Ethyl octanoate	311.31	349.98	362.04	341.11 ± 26.50 a	433.97	464.88	443.06	447.30 ± 15.89 b
1551	Ethyl nonanoate	6.07	5.88	7.46	6.47 ± 0.86 a	16.04	14.27	14.81	15.04 ± 0.91 b
1644	Ethyl decanoate	157.71	171.69	163.73	164.38 ± 7.01 a	184.92	192.41	207.43	194.92 ± 11.46 b
1695	Diethyl succinate	3.91	1.99	2.83	2.91 ± 0.96 a	2.52	1.83	3.01	2.45 ± 0.59 a
2104	Ethyl cinnamate	7.81	8.38	11.24	9.14 ± 1.84 b	4.28	5.37	4.19	4.61 ± 0.66 a
2162	Methyl vanillate	32.14	29.75	36.53	32.81 ± 3.44 b	25.53	19.86	23.2	22.86 ± 2.85 a
2240	Ethyl hexadecanoate	24.72	26.21	27.23	26.05 ± 1.26 b	15.37	16.05	18.69	16.70 ± 1.75 a
2355	Ethyl vanillate	64.82	71.31	62.33	66.15 ± 4.64 b	52.75	59.37	56.13	56.08 ± 3.31 a
	**Terpenes**								
1551	Linalool	5.9	4.57	5.67	5.38 ± 0.71 a	6.85	5.91	6.39	6.38 ± 0.47 b
1683	α-Terpineol	2.88	2.97	3.39	3.08 ± 0.27 a	4.56	5.73	4.39	4.89 ± 0.73 b
1713	Naphthalene	3.84	2.51	3.17	3.17 ± 0.67 b	2.09	2.68	2.31	2.36 ± 0.30 a
1779	β-Citronellol	1.57	1.98	2.36	1.97 ± 0.40 a	1.93	0.96	1.52	1.47 ± 0.49 a
1842	Geraniol	5.17	6.18	4.76	5.37 ± 0.73 a	7.3	8.49	8.11	7.97 ± 0.61 b
	**Phenols**								
1906	Guaiacol	325.61	358.28	302.94	328.94 ± 27.82 b	275.14	294.07	266.21	278.47 ± 14.23 a
1997	Phenol	103.61	98.78	113.44	105.28 ± 7.47 b	88.4	94.58	90.22	91.07 ± 3.18 a
2098	4-Methylphenol	7.53	8.91	6.75	7.73 ± 1.09 a	9.18	10.33	9.03	9.51 ± 0.71 b
2130	Eugenol	11.93	12.26	11.69	11.96 ± 0.29 b	7.39	8.15	7.63	7.72 ± 0.39 a
2217	4-Ethylphenol	4.49	5.91	6.37	5.59 ± 0.98 b	3.21	3.91	2.91	3.34 ± 0.51 a
2252	4-Vinylguaiacol	193.51	171.59	165.43	176.84 ± 14.76 b	121.26	139.73	112.79	124.59 ± 13.78 a
2273	2,6-Dimethoxyphenol	2.67	2.12	3.82	2.87 ± 0.87 a	2.72	2.29	2.05	2.35 ± 0.34 a
	**Acids**								
1435	Acetic acid	523.73	542.08	585.38	550.40 ± 31.66 b	440.49	486.84	410.14	445.82 ± 38.63 a
1577	2-Methylpropanoic acid	2.39	2.76	3.32	2.82 ± 0.47 a	4.58	2.97	3.19	3.58 ± 0.87 a
1643	Butanoic acid	0.79	0.68	0.94	0.80 ± 0.13 a	0.83	0.94	1.01	0.93 ± 0.09 a
1678	3-Methylbutanoic acid	0.48	0.62	0.44	0.51 ± 0.09 a	0.68	0.63	0.85	0.72 ± 0.12 b
1860	Hexanoic acid	1.14	0.99	1.37	1.17 ± 0.19 a	1.95	1.42	1.88	1.75 ± 0.29 b
2057	Octanoic acid	1.71	1.86	1.36	1.64 ± 0.26 a	1.94	1.72	2.46	2.04 ± 0.38 a
2165	Nonanoic acid	7.49	6.32	8.16	7.32 ± 0.93 a	6.05	5.76	7.34	6.38 ± 0.84 a
2298	Decanoic acid	327.04	362.69	331.39	340.37 ± 19.45 b	297.04	262.06	272.02	277.04 ± 18.02 a
2316	Hexadecanoic acid	65.15	73.38	80.92	73.15 ± 7.89 b	42.58	38.69	36.47	39.25 ± 3.09 a
	**Aromatic compounds**								
1570	Benzaldehyde	21.19	19.18	22.2	20.86 ± 1.54 a	29.96	35.33	31.59	32.29 ± 2.75 b
1650	Ehyl β-phenylacetate	6.08	4.51	5.65	5.41 ± 0.81 a	5.51	6.72	5.3	5.84 ± 0.77 a
1845	β-Phenylethyl acetate	36.7	29.76	35.64	34.03 ± 3.74 b	27.5	25.73	31.27	28.17 ± 2.83 a
1860	Benzyl alcohol	269.65	239.88	259.42	256.32 ± 15.13 a	311.64	279.65	323.63	304.97 ± 22.74 b
1879	Ethyl 3-phenylpropanoate	0.71	0.65	0.79	0.72 ± 0.07 b	0.62	0.55	0.64	0.60 ± 0.05 a
1898	β-Phenylethyl alcohol	23.81	29.29	22.33	25.14 ± 3.67 b	17.54	16.1	21.98	18.54 ± 3.06 a
2446	Benzoic acid	55.73	45.72	47.74	49.73 ± 5.29 a	42.25	35.38	46.12	41.25 ± 5.44 a
	**Lactones**								
620	Butyrolactone	62.74	58.95	74.53	65.41 ± 8.13 a	99.46	104.8	122.12	108.79 ± 11.85 b

Different letters (a, b) indicate significant differences according to the Tukey HSD test (*p* < 0.05).

## Data Availability

The original contributions presented in this study are included in the article and Supplementary Materials. Further inquiries can be directed to the corresponding authors.

## Supplementary Materials

The following supporting information can be downloaded at https://www.mdpi.com/article/10.3390/foods15040624/s1. Figure S1: Grape samples (a) and RT-PCR detection of GLRaV-3 (b). Table S1: The characteristics and physicochemical indexes for Cabernet Sauvignon berries from the disease group and the control group. Table S2. The physicochemical indexes for Cabernet Sauvignon wines from the disease group and the control group. Table S3. The phenolic acids in Cabernet Sauvignon berries of the disease group and the control group (mg/L). Table S4. The phenolic acids in Cabernet Sauvignon wines of the disease group and the control group (mg/L). Table S5. The flavanols and flavonols in Cabernet Sauvignon berries from the disease group and the control group (mg/L). Table S6. The flavanols and flavonols in Cabernet Sauvignon wines of the disease group and the control group (mg/L). Table S7: The results for sensory scores in Cabernet Sauvignon wines from the disease group and the control group.

## Abbreviations

The following abbreviations are used in this manuscript:

GLRaVs	Grapevine leaf roll-associated virus
GLRaV-3	Grapevine leaf roll-associated virus 3
EFHM	Eastern foot of the Ningxia Helan Mountains
DG	Disease group
CK	Control group
UHPLC	Ultra-high performance liquid chromatography
ESI	Electrospray ionization
TOF	Time-of-flight
HPLC	High-performance liquid chromatography
DAD	Diode array detector
HS-SPME	Headspace solid-phase microextraction
GC-MS	Gas chromatography–mass spectrometry
PCA	Principal component analysis
SBW	Single-berry weight
TD	Transverse diameter
LD	Longitudinal diameter
EGCG	Epigallocatechin gallate
EGC	Epigallocatechin
EC	Epicatechin
CAT	Catechin
WDG	Wines of the disease group
WCG	Wines of the control group
