# Supplementary material for: Effects of Grapevine Leafroll-Associated Virus 3 on the Chemical and Sensory Properties of Cabernet Sauvignon Grape and Wine
Source: Foods. 2026 Feb 9;15(4):624. doi: 10.3390/foods15040624 (PMC12939401; doi:10.3390/foods15040624)
Supplement: Supplementary file 1 [file foods-15-00624-s001.zip › foods-4135534-supplementary.pdf]

Supplementary Material

Effects of Grapevine Leafroll-associated Virus 3 on the Chemical and Sensory Properties of Cabernet Sauvignon Grape and Wine

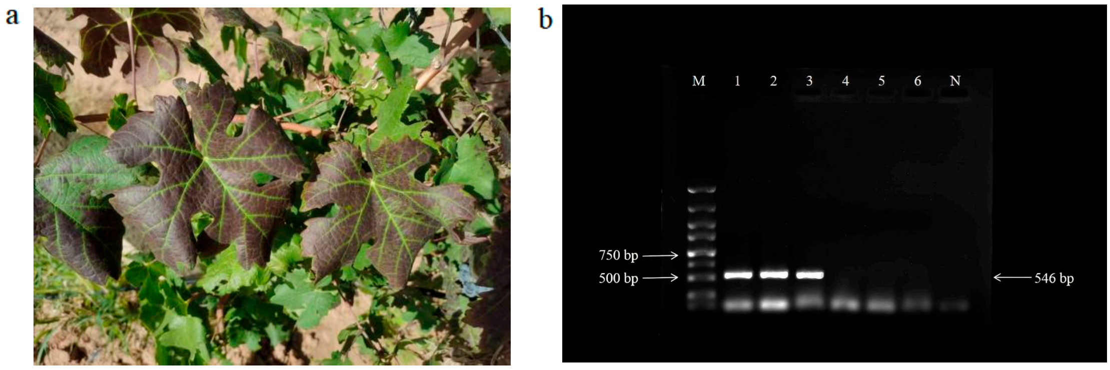

Figure S1. Grape samples (a) and RT-PCR detection of GLRaV-3 (b). (M: marker, 1-3: positive samples, 4-6: negative samples; N: negative control)

Table S1. The characteristics and physicochemical indexes for Cabernet Sauvignon berries from the disease group and the control group.

Indexes	Grapes in the disease group				Grapes in the control group			
	Sample 1	Sample 2	Sample 3	Average (SD)	Sample 1	Sample 2	Sample 3	Average (SD)
Single-grain weight (g)	1.153	1.142	1.16	1.152±0.009a	1.226	1.219	1.235	1.227±0.008b
Transverse diameters (cm)	1.128	1.092	1.184	1.135±0.046a	1.282	1.257	1.311	1.283±0.027b
Longitudinal diameters (cm)	1.13	1.19	1.082	1.134±0.054a	1.291	1.319	1.272	1.294±0.024b
Total sugars (g/L)	234.14	228.53	225.92	229.53±4.20a	240.65	239.93	235.41	238.66±2.84b
Total acids (g/L)	6.1	5.92	5.65	5.89±0.23a	5.88	5.75	5.52	5.72±0.18a
pH	3.58	3.62	3.55	3.58±0.04a	3.57	3.66	3.63	3.62±0.05b

Different letters (a, b) indicate significant differences according to the Tukey HSD test (P <0.05).

Table S2. The physicochemical indexes for Cabernet Sauvignon wines from the disease group and the control group.

Indexes	Grapes in the disease group				Grapes in the control group			
	Sample 1	Sample 2	Sample 3	Average (SD)	Sample 1	Sample 2	Sample 3	Average (SD)
Alcohol content (% vol)	13.24	13.17	13.32	13.24±0.08a	13.71	13.78	13.92	13.80±0.11b
Total sugars (g/L)	2.33	2.61	2.42	2.45±0.14a	3.20	3.14	2.86	3.07±0.18b
Total acids (g/L)	5.86	5.73	5.9	5.83±0.09b	5.61	5.69	5.71	5.67±0.05a
pH	3.69	3.68	3.7	3.69±0.01a	3.76	3.73	3.71	3.73±0.03b
Volatile acids (g/L)	0.34	0.42	0.36	0.37±0.04a	0.34	0.38	0.31	0.34±0.04a
Dry extractables (g/L)	27.19	27.63	28.05	27.62±0.43a	28.50	28.93	29.07	28.83±0.30b
Chroma	13.2	13.5	14.1	13.60±0.46a	14.34	15.35	14.89	14.86±0.50b
Hue	0.77	0.75	0.78	0.76±0.02a	0.77	0.82	0.79	0.79±0.03a

Different letters (a, b) indicate significant differences according to the Tukey HSD test (P <0.05).

Table S3. The phenolic acids in Cabernet Sauvignon berries of the disease group and the control group (mg/L).

Indexes	Grapes in the disease group				Grapes in the control group			
	Sample 1	Sample 2	Sample 3	Average (SD)	Sample 1	Sample 2	Sample 3	Average (SD)
Gallic acid	15.73	14.55	17.91	16.06±1.70a	21.98	22.47	25.01	23.15±1.63b
Protocatechuic acid	23.69	25.77	22.61	24.02±1.61b	17.29	19.55	20.31	19.05±1.57a
Gentisic acid	70.91	72.28	68.54	70.58±1.89b	42.85	46.77	44.31	44.64±1.98a
p-Hydroxybenzoic acid	13.19	13.94	12.44	13.19±0.75a	13.2	9.81	11.63	11.55±1.70a
Chlorogenic acid	11.45	10.16	8.74	10.12±1.36b	5.16	4.28	4.62	4.69±0.44a
Caffeic acid1	12.34	14.43	13.25	13.34±1.05a	19.49	17.15	18.22	18.29±1.17b
Vanillic acid	3.31	2.99	3.83	3.38±0.42a	8.58	7.2	7.89	7.89±0.69b
Syringic acid	8.18	10.35	9.01	9.18±1.09a	12.23	8.03	9.63	9.96±2.12a
p-Coumaric acid	11.18	12.64	13.91	12.58±1.37a	15.17	12.95	14.36	14.16±1.12a
Ferulic acid	11.33	9.25	10.64	10.41±1.06a	14.53	15.44	14.22	14.73±0.63b
Sinapic acid	12.21	11.63	10.72	11.52±0.75b	7.69	8.43	6.77	7.63±0.83a
Salicylic acid	177.21	185.07	202.93	188.40±13.18b	128.72	142.6	139.16	136.83±7.23a
Totals	370.73	383.06	394.53	382.77±11.90b	306.89	314.68	316.13	312.57±4.97a

Different letters (a, b) indicate significant differences according to the Tukey HSD test (P <0.05).

Table S4. The phenolic acids in Cabernet Sauvignon wines of the disease group and the control group (mg/L).

Indexes	Grapes in the disease group				Grapes in the control group			
	Sample 1	Sample 2	Sample 3	Average (SD)	Sample 1	Sample 2	Sample 3	Average (SD)
Gallic acid	35.44	37.16	30.72	34.44±3.33a	56.32	53.85	60.79	56.99±3.52b
Protocatechuic acid	15.88	17.22	13.24	15.45±2.03a	20.31	21.56	24.06	21.98±1.91b
Gentisic acid	13.65	12.97	15.33	13.98±1.21a	16.67	17.72	15.98	16.79±0.88b
p-Hydroxybenzoic acid	7.16	6.93	7.99	7.36±0.56a	6.66	7.52	5.98	6.72±0.77a
Chlorogenic acid	16.21	15.68	17.74	16.54±1.07a	19.28	20.38	21.18	20.28±0.95b
Caffeic acid	13.10	11.42	14.78	13.10±1.68a	15.39	13.76	16.02	15.06±1.17a
Vanillic acid	8.98	7.96	8.60	8.51±0.52a	6.61	8.28	7.94	7.61±0.88a
Syringic acid	14.02	12.10	11.06	12.39±1.50a	16.41	15.69	14.13	15.41±1.17b
p-Coumaric acid	3.61	2.93	3.07	3.20±0.36b	2.58	3.17	1.79	2.51±0.69a
Ferulic acid	10.05	10.77	8.91	9.91±0.94b	7.14	8.06	7.92	7.71±0.50a
Sinapic acid	8.79	7.63	7.47	7.96±0.72a	12.27	13.53	10.01	11.94±1.78b

Salicylic acid	20.54	21.41	24.28	22.08±1.97b	12.95	12.31	11.59	11.28±0.68a
Totals	167.43	164.18	163.19	164.93±2.22a	192.59	195.83	197.39	195.27±2.45b

Different letters (a, b) indicate significant differences according to the Tukey HSD test (*P* <0.05).

Table S5. The flavanols and flavonols in Cabernet Sauvignon berries from the disease group and the control group (mg/L).

Indexes	Grapes in the disease group				Grapes in the control group			
	Sample 1	Sample 2	Sample 3	Average (SD)	Sample 1	Sample 2	Sample 3	Average (SD)
Flavanols								
Epigallocatechin (EGC)	82.67	73.95	85.39	80.67±5.98a	108.94	115.08	99.86	107.96±7.66b
Catechin (CAT)	27.1	34.72	29.48	30.43±3.90a	39.33	42.19	37.47	39.66±2.38b
Epigallocatechin gallate (EGCG)	11.08	10.45	9.71	10.41±0.69b	8.46	7.51	8.61	8.19±0.60a
Epicatechin (EC)	273.18	291.01	286.55	283.58±9.28a	317.88	333.16	353.44	334.83±17.84b
Epicatechin gallate (ECG)	5.08	4.97	6.19	5.41±0.67a	7.54	9.78	10.02	9.11±1.37b
Totals	399.11	415.1	417.32	410.51±9.93a	482.15	507.72	509.4	499.76±15.27b
Flavonols								

Rutin	42.72	37.81	45.63	42.05±3.95b	36.63	33.18	35.73	35.18±1.79a
Myricaetin	14.86	15.09	13.57	14.51±0.82a	15.92	16.35	14.19	15.49±1.14a
Kaempferol	12.06	12.53	13.81	12.80±0.91b	9.84	12.21	8.48	10.18±1.89a
Quercetin	2.11	2.22	2.76	2.36±0.35b	1.35	1.2	1.58	1.38±0.19a
Totals	71.75	67.65	75.77	71.72±4.06b	63.74	62.94	59.98	62.22±1.98a

Different letters (a, b) indicate significant differences according to the Tukey HSD test (*P* <0.05).

Table S6. The flavanols and flavonols in Cabernet Sauvignon wines of the disease group and the control group (mg/L).

Indexes	Grapes in the disease group				Grapes in the control group			
	Sample 1	Sample 2	Sample 3	Average (SD)	Sample 1	Sample 2	Sample 3	Average (SD)
Flavanols								
Epigallocatechin (EGC)	192.78	179.23	186.33	186.11±6.78b	144.73	151.81	137.65	144.73±7.08a
Catechin (CAT)	436.77	405.7	487.84	443.44±41.47a	540.06	521.96	598.16	553.39±39.81b
Epicatechin (EC)	121.57	130.23	112.91	121.57±8.66b	73.23	68.79	71.67	71.23±2.25a
Totals	751.12	715.16	787.08	751.12±35.96a	758.02	742.56	807.48	769.35±33.91a

Flavonols								
Rutin	5.87	5.98	4.76	5.54±0.67a	4.07	3.91	6.23	4.74±1.30a
Myricaetin	1.53	1.86	2.10	1.83±0.29b	1.24	1.26	1.02	1.17±0.13a
Quercitrin	1.22	0.81	1.13	1.05±0.22b	0.65	0.78	0.82	0.75±0.09a
Fisetin	1.98	1.74	2.08	1.93±0.17b	1.48	1.56	1.70	1.58±0.11a
Quercetin	1.36	1.72	1.20	1.43±0.27b	0.81	0.98	1.12	0.97±0.16a
Luteolin	0.72	0.44	0.70	0.62±0.16b	0.38	0.42	0.41	0.40±0.02a
Isorhamnetin	0.64	0.45	0.53	0.54±0.10a	0.79	0.68	0.66	0.71±0.07b
Totals	13.32	13.00	12.50	12.94±0.41b	9.42	9.59	11.96	10.32±1.42a

Different letters (a, b) indicate significant differences according to the Tukey HSD test (*P* <0.05).

Table S7. The results for sensory scores in Cabernet Sauvignon wines from the disease group and the control group.

Indexes	Grapes in the disease group				Grapes in the control group			
	Sample 1	Sample 2	Sample 3	Average (SD)	Sample 1	Sample 2	Sample 3	Average (SD)
Clarity and color	9.10	9.00	9.50	9.20±0.26a	9.50	10.00	10.00	9.83±0.29b

Aroma intensity	8.61	8.54	8.73	8.63±0.10a	8.82	8.74	8.88	8.81±0.07b
Aroma complexity	8.71	8.74	8.79	8.75±0.04a	8.94	8.69	8.77	8.8±0.13a
Aroma elegance	8.34	8.25	8.41	8.33±0.08a	9.10	8.76	8.83	8.9±0.18b
Structural balance	8.57	8.49	8.63	8.56±0.07a	8.64	8.72	8.67	8.68±0.04b
Body fullness	8.45	8.46	8.37	8.43±0.05a	8.62	8.79	8.85	8.75±0.12b
Tannin quality	8.76	8.79	8.88	8.81±0.06b	8.58	8.65	8.61	8.61±0.04a
Aftertaste	8.76	8.79	8.68	8.74±0.06b	8.55	8.53	8.47	8.52±0.04a
Variety typicality	8.45	8.37	8.41	8.41±0.04a	8.66	8.74	8.83	8.74±0.09b
TOTAL	77.75	77.43	78.40	77.86±0.49a	79.41	79.62	79.91	79.65±0.25b

Different letters (a, b) indicate significant differences according to the Tukey HSD test (P <0.05).
